# Tobacco Control Policy Simulation Models: Protocol for a Systematic Methodological Review

**DOI:** 10.2196/26854

**Published:** 2021-07-26

**Authors:** Vincy Huang, Anna Head, Lirije Hyseni, Martin O'Flaherty, Iain Buchan, Simon Capewell, Chris Kypridemos

**Affiliations:** 1 Department of Public Health, Policy and Systems University of Liverpool Liverpool United Kingdom

**Keywords:** smoking, modeling, health policy, policy making, systematic review

## Abstract

**Background:**

Tobacco control models are mathematical models predicting tobacco-related outcomes in defined populations. The policy simulation model is considered as a subcategory of tobacco control models simulating the potential outcomes of tobacco control policy options. However, we could not identify any existing tool specifically designed to assess the quality of tobacco control models.

**Objective:**

The aims of this systematic methodology review are to: (1) identify best modeling practices, (2) highlight common pitfalls, and (3) develop recommendations to assess the quality of tobacco control policy simulation models. Crucially, these recommendations can empower model users to assess the quality of current and future modeling studies, potentially leading to better tobacco policy decision-making for the public. This protocol describes the planned systematic review stages, paper inclusion and exclusion criteria, data extraction, and analysis.

**Methods:**

Two reviewers searched five databases (Embase, EconLit, PsycINFO, PubMed, and CINAHL Plus) to identify eligible studies published between July 2013 and August 2019. We included papers projecting tobacco-related outcomes with a focus on tobacco control policies in any population and setting. Eligible papers were independently screened by two reviewers. The data extraction form was designed and piloted to extract model structure, data sources, transparency, validation, and other qualities. We will use a narrative synthesis to present the results by summarizing model trends, analyzing model approaches, and reporting data input and result quality. We will propose recommendations to assess the quality of tobacco control policy simulation models using the findings from this review and related literature.

**Results:**

Data collection is in progress. Results are expected to be completed and submitted for publication by April 2021.

**Conclusions:**

This systematic methodological review will summarize the best practices and pitfalls existing among tobacco control policy simulation models and present a recommendation list of a high-quality tobacco control simulation model. A more standardized and quality-assured tobacco control policy simulation model will benefit modelers, policymakers, and the public on both model building and decision making.

**Trial Registration:**

PROSPERO International Prospective Register of Systematic Reviews CRD42020178146; https://www.crd.york.ac.uk/prospero/display_record.php?ID=CRD42020178146

**International Registered Report Identifier (IRRID):**

DERR1-10.2196/26854

## Introduction

Smoking remains a top public health priority, globally killing over 6 million people annually, with 450,000 smoking-related hospital admissions, representing 4% of annual adult admissions, in England [1,2]. Furthermore, smoking prevalence demonstrates worrying inequalities, reaching 25% among routine and manual workers but only 10% among those in managerial and professional occupations [3]. Future tobacco control policies will thus need to be both effective and equitable, and might therefore greatly benefit from useful simulation models for tobacco control. Using simulation modeling to tackle complex public health issues was also highlighted in the Chief Medical Officer’s Report of 2018 [4].

Simulation models are mathematical frameworks estimating the potential impact of health care interventions, which are widely used in informing medical decision-making [5-8]. These models are commonly used in economics, transport, business, and meteorology, but less so in public health [5].

Tobacco control models, mathematical models that predict tobacco-related outcomes in defined populations, have attracted increased interest in recent years [9-11]. However, few reviews have systematically studied this topic. Feirman et al [10] published what is considered to be the first systematic review on models in the tobacco control field. They reviewed 263 studies published before July 1, 2013, and noted a diversity of model methods and applications. In general, the models aimed at projecting tobacco-related trends and policy/intervention effects with outcomes of behavior change, health effect, or economic impact. Unsurprisingly, similar to other medical decision-making models, tobacco control models are developed using diverse methods such as Markov chains, discrete event, and microsimulation. Berg et al [11] studied economics models used specifically in smoking cessation, and reported the state-transition Markov model as the most common model type, with quality-adjusted life years being the most common outcome used for assessments.

We define policy simulation models as models that estimate and compare the potential impact of existing or not-yet-implemented policies. The impacts can be health-related, equity-related, economic, environmental, or other [5]. Therefore, models potentially represent the best methodological approach for estimating the future benefits of diverse prevention policies [12]. Nevertheless, some model audiences and potential users remain concerned about model credibility. As stated by the Brighton Declaration, model transparency and reporting guidelines are major existing challenges [5]. Similarly, the International Society for Pharmacoeconomics and Outcomes Research (ISPOR)-Society for Medical Decision Making (SMDM) Modelling Good Research Practice Task Force emphasized the role of transparency in explaining how the models work and the importance of validity in demonstrating model accuracy [6,7,13,14].

Quality assessment is a strategy used in weighing the credibility of study findings [15]. There are several publicly available quality assessment tools, including the Consolidated Health Economic Evaluation Reporting Standards (CHEERS) checklist; Grading of Recommendations, Assessment, Development and Evaluations (GRADE); and the National Institute for Health and Care Excellence (NICE) Methodology Guide quality checklist. However, these checklists are not all applicable to the evaluation of tobacco control models. The NICE and CHEERS checklists are designed for evaluation of economics models, and the GRADE guideline mainly focuses on evidence certainty [15-17]. Neither of the previous tobacco control model reviews applied a quality assessment of model transparency, validation, or reporting standard. Feirman et al [10] did not assess study quality owing to high heterogeneity among studies. Similarly, Berg et al [11] only evaluated study limitations and economic parameters. Nevertheless, both papers discussed the importance of reporting quality on model process and output, thus highlighting the need for further research on model transparency, validation, and reporting quality.

Tobacco control policy simulation models, as a policy model subcategory of tobacco control models, is an active area of research. In this systematic review, we will update, expand, and enhance the work of Feirman et al [10]. Specifically, we will perform a systematic methodological review on tobacco control policy simulation models to (1) assess the modeling practices used in tobacco control policy simulation models, and (2) present a recommendation list of a high-quality tobacco control policy simulation model. Model users will be able to evaluate tobacco control models using this recommendation, which will better enable decision makers with tobacco policy decision making.

## Methods

### Study Design

We will perform a systematic methodological review of tobacco control policy simulation models following the Preferred Reporting Items for Systematic Reviews and Meta-Analysis (PRISMA) checklist to ensure proper conduct. This checklist offers a systematic way to search each database, minimizing the impact of the researcher on the outcome of the search [18]. We will use a narrative synthesis to present the data.

### Search Strategies

We expanded a search strategy from a peer-reviewed systematic review of population tobacco use prediction models to identify potential literature [9]. Five electronic bibliographic databases (Embase, EconLit, PsycINFO, PubMed, and CINAHL Plus) were searched. A sample of search terms used in PubMed is provided in Textbox 1; the full search terms are provided in Multimedia Appendix 1. The final search was performed on August 1, 2019 limited to the English language and publication date from July 2013 to August 2019. Papers identified by the searches were imported into Zotero (version 5.0.85), a data management program, to identify duplicates, and screen titles, abstracts, and full texts as appropriate.

**Sample of search terms used in PubMed.**

((“models, theoretical”[majr:noexp] OR “models, statistical”[majr:noexp] OR “models, economic”[majr] OR “computer simulation”[majr:noexp] OR “monte carlo method”[mesh] OR “decision support techniques”[majr:noexp] OR “decision trees”[mesh] OR “systems theory”[mesh] OR “markov chains”[mesh] OR “system dynamics”[tiab] OR “agent-based model”[tiab] OR “agent-based models”[tiab] OR “agent-based modelling”[tiab] OR “agent-based modelling”[tiab] OR “simulation model”[tiab] OR “decision analysis”[tiab] OR “decision framework”[tiab] OR “markov”[tiab] OR “cost-utility analysis”[tiab] OR “cost-utility analyses”[tiab] OR “cost-effectiveness analysis”[tiab] OR “cost-effectiveness analyses”[tiab] OR “cost-benefit analysis”[tiab] OR “cost-benefit analyses”[tiab] OR “forecasting”[mesh] OR “microsimulation”[tiab] OR “micro simulation”[tiab] OR “monte carlo” [tiab] OR “life year”[tiab] OR “life years”[tiab] OR “smoking-attributable deaths”[tiab] OR “smoking attributable deaths”[tiab] OR “deterministic”[tiab] OR “probabilistic”[tiab] OR “stochastic”[tiab] OR “dynamic transmission model”[tiab] OR “state-transition”[tiab] OR “state transition”[tiab] OR “discrete event”[tiab] OR “continuous event”[tiab] OR “analytic horizon”[tiab] OR “cohort simulation”[tiab] OR “second-order simulation”[tiab] OR “threshold analysis”[tiab] OR “years of healthy life”[tiab] OR “decision problem”[tiab] OR “transition probabilities”[tiab] OR “discount rate”[tiab]) AND (“Smoking”[Mesh] OR “Smoking Cessation”[Mesh] OR “Tobacco”[Mesh] OR “Tobacco Products”[Mesh] OR “Tobacco, Smokeless”[Mesh] OR “Smoking”[TI] OR “Tobacco”[TI] OR “Smoker”[TI] OR “Smokers”[TI] OR (cigar[TI] OR cigar'[TI] OR cigarettes[TI] OR cigaret[TI] OR cigarete[TI] OR cigarets[TI] OR cigarett[TI] OR cigarette[TI] OR cigarette'[TI] OR cigarette's[TI] OR cigarettedagger[TI] OR cigaretteinduced[TI] OR cigarettes[TI] OR cigarettes'[TI] OR cigarettesmoke[TI] OR cigaretts[TI] OR cigarillo[TI] OR cigarillos[TI] OR cigarlike[TI] OR cigarra[TI] OR cigarret[TI] OR cigarrette[TI] OR cigarrilla[TI] OR cigarro[TI] OR cigarros[TI] OR cigars[TI]) OR “Smokeless”[TIAB] OR (e cigarette[TIAB] OR e cigarette's[TIAB] OR e cigarettedagger[TIAB] OR e cigarettee[TIAB] OR e cigarettes[TIAB]) OR (electronic cigarette[TIAB] OR electronic cigarettes[TIAB]) OR “Snus”[TIAB] OR “Nicotine”[TIAB]))

### Study Selection and Inclusion Criteria

Studies were included when they contained peer-reviewed tobacco control policy simulation models that predict tobacco-related outcomes from smoking policy options and scenarios. We are interested in modeling the methodologies of tobacco control policy simulation models across a variety of population groups; therefore, we will include tobacco control policy simulation models with any subpopulation in any setting.

The retrieved studies were assessed using the PICOS (Participants, Interventions, Comparators, Outcomes, and Study design) approach (Table 1). Two reviewers (VH and AH) independently assessed the eligibility of the studies. Any discrepancies were resolved by consensus or by involving the senior author (CK).

**Table 1:** PICOS (Participants, Interventions, Comparators, Outcomes, and Study design) approach to set inclusion and exclusion criteria.

Category	Include	Exclude
Participants	Studies on any populations	Studies on animals and cells
Interventions	Tobacco control policies	Nontobacco control policies
Comparator	Studies where tobacco control policy simulation models are evaluated or compared	No tobacco control policy simulation models presented
Outcomes	Studies reporting any tobacco-related outcomes	Studies reporting no tobacco-related outcomes
Study design	Policy simulation models	Studies without policy simulation models

### Data Extraction

A data extraction form facilitates the extraction of bibliographic and methodological information about each study, and ensures that data extraction is consistent among all reviewers and across all studies. Use of such a form could also aid subsequent analyses [19].

Three reviewers (VH, AH, and CK) designed a data extraction form based on our research questions, referring to existing guidelines and expert opinions. The form has already been piloted in several studies that will be included in this systematic review. The form will be used to collect thorough information on model structure, data sources, and transparency, including the following categories (see Multimedia Appendix 2 for the full extraction form): (1) general model information (eg, model name, code license, conflict of interest); (2) model simulation methods (eg, model type); (3) demographic characteristics (eg, age, gender, ethnicity/race, socioeconomic status); (4) risk factors; (5) diseases; (6) data sources; (7) model outcome types (eg, health, economics); (8) model checking (model transparency and validation/calibration); (9) reported model limitations. All data will be extracted into Microsoft Excel.

Reviewers VH and AH will independently perform data extraction on included studies. Each study will be extracted by only one reviewer. To ensure consistency, the reviewers will discuss and compare data extraction after reviewing five studies. When any unclear, missing, or insufficient data are encountered, the reviewers will contact the study authors for clarification.

### Quality Assessment

We are not aware of any widely accepted quality assessment for policy simulation models. To elaborate, previous tobacco control policy simulation model review papers did not perform any quality assessment owing to study heterogeneity or a different study purpose.

Therefore, using the findings of our review, we are aiming to describe the ideal high-quality tobacco control policy simulation model. To be specific, we will employ criteria regarding the quality of (a) model inputs (hierarchy of evidence); (b) model structure (population representativeness, exposure granularity, disease epidemiology); and (c) model outputs (reporting standards, uncertainty and sensitivity analysis, model validation) to analyze current models. We are aiming to use this model quality standard to facilitate future discussions on a policy simulation model quality assessment framework.

### Data Synthesis

First, individual studies will be grouped by model names or common author names, as there are models that have been used in more than one published study. We will report patterns and trends related to modeling methods, outcome types, and funding sources (if the study is industry-funded). As modeling is an evidence-synthesis methodology, we will study the synthesis methods used among models by dissecting their approaches. We will critically review model data inputs, epidemiological principles, assumptions, and transparency. Moreover, we will identify the best practices and common pitfalls shared by identified models. Synthesizing the findings, we will provide a recommended list of the elements that a high-quality tobacco control policy simulation model should have. The model reporting quality will also be analyzed according to the criteria described in the Quality Assessment section above.

We will present our findings in complementary graphical formats using tables and charts.

## Results

We are currently in the data collection stage. We are expecting to complete and submit our results for publication by April 2021.

## Discussion

To the best of our knowledge, this is the first systematic review to focus on tobacco control policy simulation models. As well as summarizing model best practices and pitfalls, we will advise on the quality assessment of tobacco control policy simulation models. This assessment will be informed by referring to our study results, the published literature, and workshop reports published by the ISPOR-SMDM Modelling Good Research Practice Task Force and other relevant organizations.

This research could benefit modelers, policymakers, and the public from various backgrounds. The development and dissemination of a model framework, and the identification of best practices and weaknesses for model development may serve as a useful resource for future modelers to improve current models and plan more advanced and high-quality models. Our model reporting statement will enable the improvement of model credibility by emphasizing model reporting standards on transparency and validity.

Policymakers and journal editors may appreciate our characterization of a high-quality model. Furthermore, this work may help policymakers make quicker and more accurate decisions on model selection and model outcome evaluation, which will also ultimately benefit patients and the public.

Last but not least, reinforcing ISPOR-SMDM Modelling Good Research Practice Task Force principles, our results may help kickstart a more standardized and quality-assured tobacco control policy simulation model era. This could also inspire modelers working in other fields to enhance model quality.

This protocol for a systematic methodological review has several limitations. First, our search result is limited by publication language due to resource limits. However, there could be further research incorporating papers written in other languages to compare and verify our research findings. Moreover, we only searched and analyzed papers published from July 2013 to August 2019 in this study. We could expand our data extraction and synthesis to tobacco control policy simulation models identified in the study from Feirman and colleagues [10].

## Appendix

Multimedia Appendix 1 Search strategy.

Multimedia Appendix 2 Data extraction form.

## Abbreviations

CHEERS: Consolidated Health Economic Evaluation Reporting Standards
GRADE: Grading of Recommendations, Assessment, Development and Evaluations
ISPOR: International Society for Pharmacoeconomics and Outcomes Research
NICE: National Institute for Health and Care Excellence
PICOS: Participants, Interventions, Comparators, Outcomes, and Study design
PRISMA: Preferred Reporting Items for Systematic Reviews and Meta-Analysis
SMDM: Society for Medical Decision Making
