# Supplementary material for: Tobacco Control Policy Simulation Models: Protocol for a Systematic Methodological Review
Source: JMIR Res Protoc. 2021 Jul 26;10(7):e26854. doi: 10.2196/26854 (PMC8367099; doi:10.2196/26854)
Supplement: Multimedia Appendix 2 [file resprot_v10i7e26854_app2.docx]

**Multimedia Appendix 2.** Data extraction form.

| Paper Name |  |
| --- | --- |
| 1. GENERAL INFORMATION | |
| Paper author (First author) |  |
| Paper published year (published online) |  |
| Ref ID (DOI): |  |
| Data extractor: |  |
| Extraction date (DD/MM/YYYY) |  |
| Funding & Conflict of interest |  |
| General information - Others |  |
| 2. MODEL DETAILS | |
| Model name |  |
| Code license/ Open source |  |
| code URL |  |
| Model setting - Country/Area |  |
| Model - Initial year |  |
| Prediction period: |  |
| model detail - others |  |
| 3. TYPE OF MODEL | |
| Agent based model |  |
| Decision tree |  |
| Discrete event |  |
| Life table |  |
| Markov model |  |
| Macrosimulation |  |
| Microsimulation |  |
| System dynamic |  |
| Open cohort |  |
| Close cohort: |  |
| Continuous time |  |
| Discrete time |  |
| Type of model - others |  |
| 4. DEMOGRAPHIC CHARACTERISTICS (Tick if applicable) | |
| Gender (Y, both, F, M) |  |
| Age |  |
| Socioeconomic status |  |
| Education |  |
| Income |  |
| Race/ Ethnicity |  |
| Urban/ Rural |  |
| Demographic - Others |  |
| 5. RISK FACTORS | |
| Alcohol intake |  |
| Alcohol intake (Unit) |  |
| Blood pressure |  |
| Blood pressure (Unit) |  |
| Cholesterol |  |
| Cholesterol (Unit) |  |
| Competing causes |  |
| Competing causes (Unit) |  |
| Diabetes |  |
| Diabetes (Unit) |  |
| Environmental tobacco smoking |  |
| Environmental tobacco smoking (Unit) |  |
| Fruit and vegetable consumption |  |
| Fruit and vegetable consumption (Unit) |  |
| General Health status |  |
| General Health status (Unit) |  |
| Hypertension |  |
| Hypertension (Unit) |  |
| Mental health |  |
| Mental health (Unit) |  |
| Obesity or BMI |  |
| Obesity or BMI (Unit) |  |
| Physical activity |  |
| Physical activity (Unit) |  |
| Other risk factors (list down in box) |  |
| Other risk factors (list down in box) (Unit) |  |
| Smoking Status (never, former, smoker) (Unit) |  |
| Smoking status (Unit) |  |
| Smoking history (age star/ duration, intensity/age quit) |  |
| Unit (pack- year, smoking duration, smoking intensity, smoking duration and intensity independently) |  |
| Lag time |  |
| Lag time (Unit) |  |
| Risk factor-others |  |
| Risk factor-others (Unit) |  |
| 6. OUTCOME TYPE | |
| Equality |  |
| Economics outcome |  |
| Hospital admission |  |
| Health outcomes - mortality |  |
| Health outcomes - morbidity |  |
| Health outcomes - other |  |
| Smoking attitude/ Smoking prevalence |  |
| Uncertainty |  |
| Outcome types - Others (please describe) |  |
| 7. DISEASE CATEGORIES | |
| AMI (Acute myocardial infarction) |  |
| Atrial fibrillation (AF) |  |
| Asthma |  |
| COPD |  |
| CVD |  |
| Diabetes |  |
| Diabetic neuropathy |  |
| Diabetic retinopathy |  |
| Dyslipidaemia |  |
| Lung cancer |  |
| Obesity |  |
| Other cancers |  |
| Stroke |  |
| Tuberculosis (TB) |  |
| Hypertension |  |
| Diseases - Others |  |
| Disease categories - others |  |
| 8. DATA SOURCES USED | |
| Population |  |
| Mortality |  |
| Morbidity |  |
| Policy effective/ treatment effectiveness |  |
| Data source - Others |  |
| 9. MODEL CHECKING | |
| Any sensitivity analyses carried out? |  |
| Which sensitivity analyses were carried out? |  |
| Was the model aligned? |  |
| Was the model calibrated? |  |
| How was the model calibrated? |  |
| Was the validity of the model tested? |  |
| Face validation |  |
| Internal validation |  |
| Cross validation |  |
| External validation |  |
| Validation - others |  |
|  | |
| Nontechnical & Technical documentation |  |
| Assumptions |  |
| Model availability for reader (not including source code) |  |
| Transparency - others |  |
| Model checking - others |  |
| 10. POTENTIAL LIMITATIONS | |
| Please list down Limitation |  |
